# Effective fractionation of lignocellulose components and lignin valorization by combination of deep eutectic solvent with ethanol

**DOI:** 10.3389/fbioe.2022.1115469

**Published:** 2023-01-09

**Authors:** Pingping Cui, Zhishang Ye, Mengzhen Chai, Jie Yuan, Yan Xiong, Haitao Yang, Lan Yao

**Affiliations:** ^1^ Hubei Provincial Key Laboratory of Green Materials for Light Industry, Hubei University of Technology, Wuhan, China; ^2^ State Key Laboratory of Biobased Material and Green Papermaking, Shandong Academy of Sciences, Qilu University of Technology, Jinan, Shandong, China; ^3^ Key Laboratory of Fermentation Engineering (Ministry of Education), Cooperative Innovation Center of Industrial Fermentation (Ministry of Education and Hubei Province), College of Bioengineering, Hubei University of Technology, Wuhan, China

**Keywords:** DES, pretreatment, enzymatic saccharification, lignin, antioxidant

## Abstract

**Introduction:** A combination of deep eutectic solvent with ethanol was developed for pretreatment of *Broussonetia papyrifera* to effectively extract lignin and promote the subsequent enzymatic hydrolysis.

**Methods:** In order to further explore the optimal conditions for enzymatic hydrolysis, a central composite design method was applied.

**Results and Discussion:** The correlation between each factor and glucose yield was obtained, and the optimal conditions was 160°C, 60 min, the ratio of DES to E was 1/1 (mol/mol). The results showed that compared with control, the glucose yield increased by 130.67% under the optimal pretreatment conditions. Furthermore, the specific surface area of biomass was increased by 66.95%, and the content of xylan and lignin was decreased by 86.71% and 85.83%. The correlation between xylan/lignin removal and enzymatic hydrolysis showed that the removal of lignin facilitated the glucose yield more significantly than that of xylan. To further explore the lignin valorization, the structural and antioxidant analysis of recovered lignin revealed that high temperature was favorable for lignin with good antioxidant performance. This pretreatment is a promising method for separating lignin with high antioxidant activity and improving cellulose digestibility.

## 1 Introduction

In recent years, the sharp increase in the consumption of fossil fuels results in the rise of crude oil prices and serious environmental problems. As a kind of green energy, renewable energy can replace fossil fuel, which is widely concerned worldwide (Thi and Lee, 2019; Peng et al., 2021). One of them is bioethanol derived from lignocellulose. The composition of lignocellulose biomass is complex, which includes carbohydrate (cellulose, hemicellulose) and aromatic polymer (lignin) (Wang Y. et al., 2022). In the plant cell wall, hemicellulose and lignin are connected by covalent bonds and hydrogen bonds to form heterogenous structure, which is coated on cellulose to form is a strong “natural anti degradation barrier”. This barrier greatly limits the efficiency of biomass refinery (Penín et al., 2020). Pretreatment is an essential process to overcome the recalcitrance of biomass and achieve efficient fractionation of biomass components.

Deep eutectic solvents (DESs) are composed of hydrogen bond receptors (HBAs) and hydrogen bond donors (HBDs), which contain large and asymmetric ions, resulting in low lattice energy and therefore low melting point (Gontrani et al., 2019; Xu et al., 2020; Yu et al., 2021). Because DES could form rich hydrogen bonds, it has good solvability for polar and non-polar compounds (Pandey et al., 2017). DES pretreatment has achieved remarkable results in biomass fractionation and digestibility improvement (Hou et al., 2022). The cellulose conversion of DES pretreated corn stover and corncob exceeds 90% (Xu et al., 2016; Zhang et al., 2016; Hou et al., 2017). Furthermore, DES shows advantages of simple preparation, low toxicity, recyclability and biodegradability, thus wide application potential in the utilization of lignocellulose (Bu et al., 2021; Wang W. et al., 2022).

DES based on choline chloride (ChCL) can effectively break linkages within lignin carbohydrate complex (LCC) (Shen et al., 2019; 2021; Zhou et al., 2021). It was found that the addition of the third HBD component to the ChCL based DES can change fluidity and thermal stability of DES, thus improving the pretreatment performance of biomass (Xia et al., 2018). The most widely applied DES is composed of ChCL/LA (lactic acid). Earlier research showed that lignin re condensed during pretreatment (Bjelic et al., 2022). In our previous research, it was found that DES with ethanol was more efficient in digestibility improvement than ChCL/LA (Yao et al., 2022). However, the previous research on the optimization of pretreatment conditions is still incomplete.

Lignin is an important component that needs to be valorized to obtain total biomass utilization for biorefinery (Wang et al., 2022). Lignin itself has different kinds of active functional groups, which has advantages in scavenging free radicals. Therefore, it is of great interest to explore the value-added utilization of lignin. Organosolv lignins are the most studied one as antioxidant. Our previous research showed that physicochemical properties had great impact on the capability of scavenging free radicals (Yao et al., 2020). To be specific, the contents of phenolic hydroxyl groups had positive effects on the antioxidant activity of lignin. To further explore the impact of DES-E pretreatment on recovered lignin and its valorization, more research needs to be done. In this study, DES with ethanol was synthesized with choline chloride lactic acid and ethanol. The central composite design method was employed to obtain the best pretreatment conditions (mol ratio of DES to ethanol, temperature and time) to promote the enzymatic hydrolysis and saccharification of pretreated biomass. Furthermore, the structural properties and antioxidant activity of recovered lignin after pretreatment were analyzed. The relationship between the structural properties of lignin and its antioxidant activity was studied.

## 2 Material and methods

### 2.1 Materials

*Broussonetia papyrifera* was given by a local farmer in Wuhan, Hubei Province. After size reduction to 60–80 meshes, it was extracted with benzene-alcohol for 6 h. The component analysis results showed that the content of glucan, xylan, lignin and ash was 36.31%, 27.89%, 19.99%, and 1.01% respectively. Cellulase is provided by Baiyin Sainuo Technology Co., Ltd. (Gansu Province, China), with 160U/g filter paper activity. All other chemical reagents were purchased from Sigma without further purification.

### 2.2 Preparation of DES

After vacuum oven dried at 50°C for 24 h, lactic acid and choline chloride was mixed in a round bottom flask (2:1 M ratio) in an oil bath at 90°C for 2 h to form a uniform, stable and transparent liquid. After cooling to room temperature, it was vacuum oven dried for 1 week before use.

### 2.3 Pretreatment and lignin isolation

50 g dried wood powder and 500 g DES-E were mixed into a 1-L rotary electrothermal pressure digester (Parr Instrument Company). After keeping at a certain temperature for a period of time, the pretreated liquid and biomass was separated by filtration. The solid residues were first washed with excess ethanol to remove DES, and then washed with deionized water until the pH was neutral. The pretreated solid residues were stored at 4°C for further cellulase saccharification. In order to obtain the optimal pretreatment conditions, pretreatment temperature, time, and the ratio of DES to ethanol were optimized by central composite design (CCD). The statistical analysis software ‘‘Statistica 10.0” and ‘‘Minitab” (Minitab Inc.) were used for the experimental design and data analysis (*p*-value analysis), respectively. Pretreatment yield, component analysis and removal rate was calculated as follows:

$$Pretreatment\;yield=\frac{\begin{array}{c} oven\;dried\;biomass\;weight\\ \;after\;pretreatment \end{array}}{\begin{array}{c} oven\;dried\;biomass\;weight\\ \;before\;pretreatment \end{array}}\times 100\%$$ (1)

$$Cellulose\;removal=1-\frac{\begin{array}{c} Pretreatment\;yield\times \\ Cellulose\;amount\;in\;\\ residue\;after\;pretreatment \end{array}}{\begin{array}{c} cellulose\;amount\;\\ \;before\;pretreatment \end{array}}\times 100\%$$ (2)

$$Xylan\;removal=1-\frac{\begin{array}{c} Pretreatment\;yield\times Xylan\;amount\;\\ in\;residue\;after\;pretreatment \end{array}}{Xylan\;amount\;before\;pretreatment}\times 100\%$$ (3)

$$Lignin\;removal=1-\frac{\begin{array}{c} Pretreatment\;yield\times Lignin\;amount\;\\ in\;residue\;after\;pretreatment \end{array}}{Lignin\;amount\;before\;pretreatment}\times 100\%$$ (4)

**FIGURE 1:**
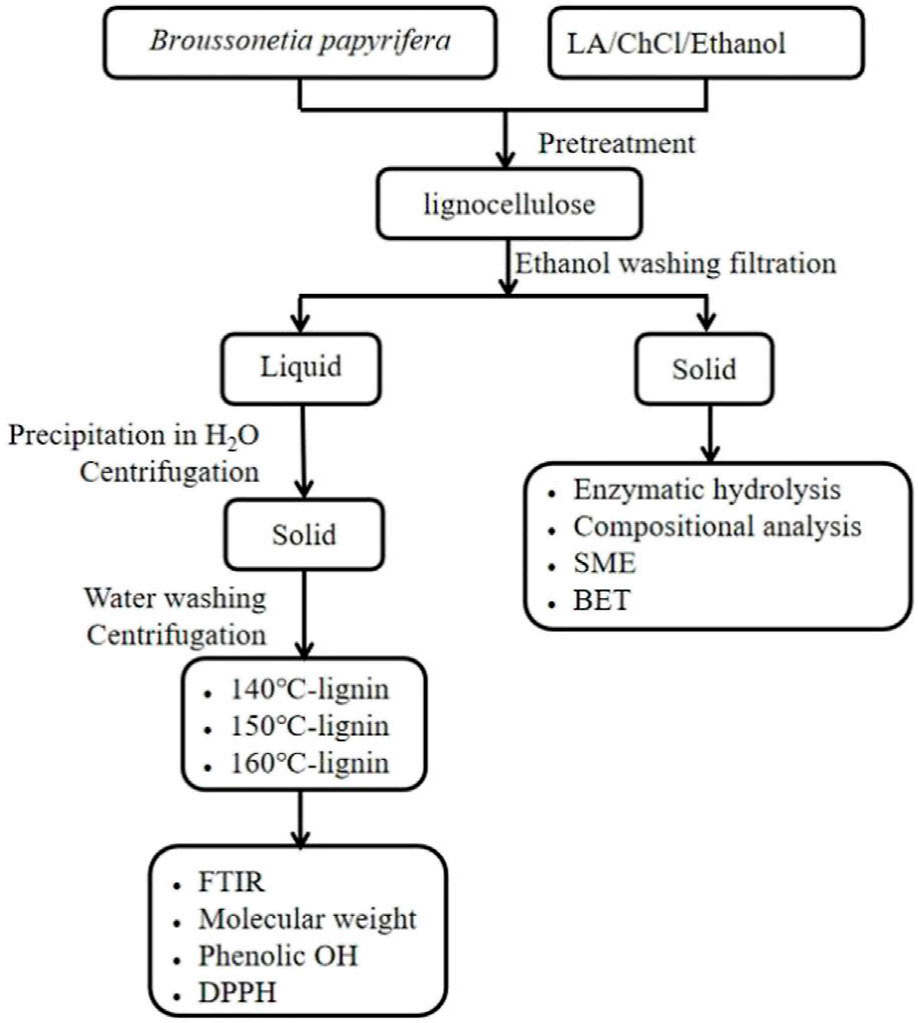
Flow chart of the pretreatment process.

After pretreatment, lignin rich liquid fractions were concentrated by rotary evaporation. The recovered lignin was then obtained by precipitating in excess amount of cold DI water. The precipitated lignins (140°C—lignin, 150°C—lignin, and 160°C—lignin) were obtained by centrifugation, washed with DI water and 90% ethanol, and finally freeze-dried, as shown in Figure 1.

### 2.4 Enzymatic hydrolysis

Pretreated *Broussonetia papyrifera* were enzymatically hydrolyzed with cellulase. The enzyme loading was 25 FPU/g, and the solid concentration was 2%. The enzyme hydrolysis was conducted at 50°C for 72 h. The glucose content was analyzed by HPLC (Shimadzu, Kyoto, Japan) with a refractive index detector (Shimadzu) on an Aminex HPX-87P column (Bio-Rad, Hercules, CA, United States), with water as eluent. The cellulose digestibility and glucose yield were calculated as follows:

$$Cellulose\;Digestibility=\frac{\begin{array}{c} Cellulose\;amount\;in\;\\ enzymatic\;hydrolysis\;solution \end{array}}{Cellulose\;amount\;in\;residue}\times 100\%$$ (5)

$$Glucose\;Yield=Pretreatment\;yield\times \frac{\begin{array}{c} glucose\;amount\;in\;\\ enzymatic\;hydrolysis\;\\ solution\times 0.9 \end{array}}{\begin{array}{c} Cellulose\;amount\;\\ \;in\;residue \end{array}}\times 100\%$$ (6)

### 2.5 SEM and BET determination of pretreated biomass

Scanning electron microscope (SU8010; HITACHI Ltd., Tokyo, Japan) was applied to observe the fiber morphology under 5 kV accelerating voltage.

The surface of pretreated biomass was determined by nitrogen adsorption method. The adsorption/desorption isotherms of nitrogen at 77 K were measured applying a BELSORP mini-II surface area and pore size analyzer (MicrotracBEL, Japan).

### 2.6 Determination of physical and chemical properties of recovered lignin

FT-IR 710 infrared spectrophotometer was employed for FTIR analysis. The Gel permeation chromatography was applied to determine the molecular weight of lignin (LC-10AD, Shimadzu Co., Ltd., Japan) after being acetylated (Kumar et al., 2013). Phenolic hydroxyl group content was determined by ultraviolet spectrophotometer (UV-2550, Shimadzu Co., Ltd., Japan) (Tan et al., 2019).

### 2.7 Determination of antioxidant activity by DPPH method

The antioxidant activity of lignin was determined by 1,1-diphenyl-2-trinitrophenylhydrazine (DPPH) method. Briefly, lignin was dissolved in 90% dioxane water (v/v) solution with different concentrations. Then lignin was mixed with DPPH solution in dark for 1 h, after which the ultraviolet absorption value of each solution was determined at 517 nm. The free radical scavenging activity and antioxidant activity indexes were calculated according to previous study (Sadeghifar et al., 2017).

## 3 Results and discussion

### 3.1 Optimization of pretreatment condition by response surface method

Different DESs (various mol ratios) were synthesized, and their performance in improving glucose yield of *Broussonetia papyrifera* at different temperature and time was evaluated. Based on the preliminary results, three factors (mol ratio of DES to E, temperature, time) were optimized by central composite design. The pretreatment conditions, cellulose digestibility and glucose yield were shown in Table 1.

**TABLE 1:** Experimental design and results of central composite design.

	Ratio of DES to E (mol·mol^−1^)	Temperature (°C)	Time (h)	Pretreatment	Cellulose	Glucose
				Yield (%)	Digestibility (%)	Yield (%)
Untreated	—	—	—	—	20.05 ± 1.18	20.05 ± 1.18
1	0.5	150	0.5	71.33 ± 0.43	27.01 ± 3.44	19.26 ± 0.68
2	2	150	0.5	63.36 ± 0.69	50.78 ± 0.55	32.17 ± 1.26
3	0.5	150	1.5	51.94 ± 1.06	69.85 ± 2.31	36.28 ± 1.17
4	2	150	1.5	47.02 ± 0.20	80.42 ± 2.96	37.81 ± 0.07
5	0.5	170	0.5	48.97 ± 0.20	73.73 ± 5.90	36.11 ± 0.20
6	2	170	0.5	42.89 ± 0.20	74.49 ± 1.89	31.95 ± 0.20
7	0.5	170	1.5	42.05 ± 0.20	75.71 ± 0.57	31.83 ± 0.20
8	2	170	1.5	39.85 ± 0.02	96.43 ± 3.05	38.43 ± 0.15
9	0.5	160	1	52.56 ± 1.09	83.31 ± 1.95	43.78 ± 0.91
10	2	160	1	46.06 ± 1.53	91.11 ± 1.10	41.98 ± 1.63
11	1	150	1	63.11 ± 5.63	66.27 ± 2.13	41.75 ± 1.70
12	1	170	1	45.15 ± 1.75	97.57 ± 0.67	44.03 ± 0.99
13	1	160	0.5	56.91 ± 0.43	64.61 ± 1.59	36.77 ± 0.68
14	1	160	1.5	46.25 ± 1.06	94.27 ± 4.28	43.57 ± 1.17
15	1	160	1	49.45 ± 0.69	93.52 ± 2.18	46.25 ± 1.26

Trials No. 9, 15, and 10 were conducted with the same pretreatment temperature and time, and the molar ratio of DES to ethanol was 1/2, 1/1 and 2/1, respectively. The results showed that pretreatment yield was decreased with the enhanced DES content (52.56%, 49.45%, and 46.06%), indicating that more DES could dissolve more biomass during pretreatment. The cellulose digestibility (83.31%, 93.52%, and 91.11%) and the glucose yield (43.78%, 46.25%, and 41.98%) was increased first and then decreased with more of DES content. The results showed that more DES loading did not favor enzymatic hydrolysis of pretreated *Broussonetia papyrifera*. This may be due to that the excessive removal of lignin and xylan, which led to the collapse of cellulose structure and the reduction of the available surface area of cellulose for cellulase adsorption (Shen et al., 2019).

*Broussonetia papyrifera* were pretreated at the same DES to E ratio and time at 150°C, 160°C, and 170°C in scheme No 11, 15, and 12, respectively. It was obvious to see that solid residue yield was decreased at higher pretreatment temperature (63.11%, 49.45%, and 45.15%). The cellulose digestibility was 66.27%, 93.52%, and 97.57%, respectively, indicating that the increase of pretreatment temperature was in favor of cellulose conversion. Earlier literature showed that the cellulose conversion of pretreated poplars with DES was significantly increased with the increase of pretreatment temperature (Yang et al., 2021). Due to the decreased pretreatment yield with increased pretreatment temperature, the glucose yield was increased first and then decreased (41.75%, 46.25%, and 44.03%).

Scheme No 13, 15, and 14 were pretreated with the same DES to E ratio and temperature, but with various time (0.5, 1, and 1.5 h). Results showed that the pretreatment yield was decreased with extension of time (56.91%, 49.45%, and 46.25%). After pretreatment with DES-E, the ether linkage in lignin carbohydrate complex (LCC) was broken (Li et al., 2022). With the extension of pretreatment time, more surfaces and pores were exposed for cellulase, thus increasing the cellulose digestibility (64.61%, 93.52%, and 94.27%). Taking pretreatment yield into consideration, glucose yield was first increased and then decreased with extended time (36.77%, 46.25%, and 43.57%).

**FIGURE 2:**
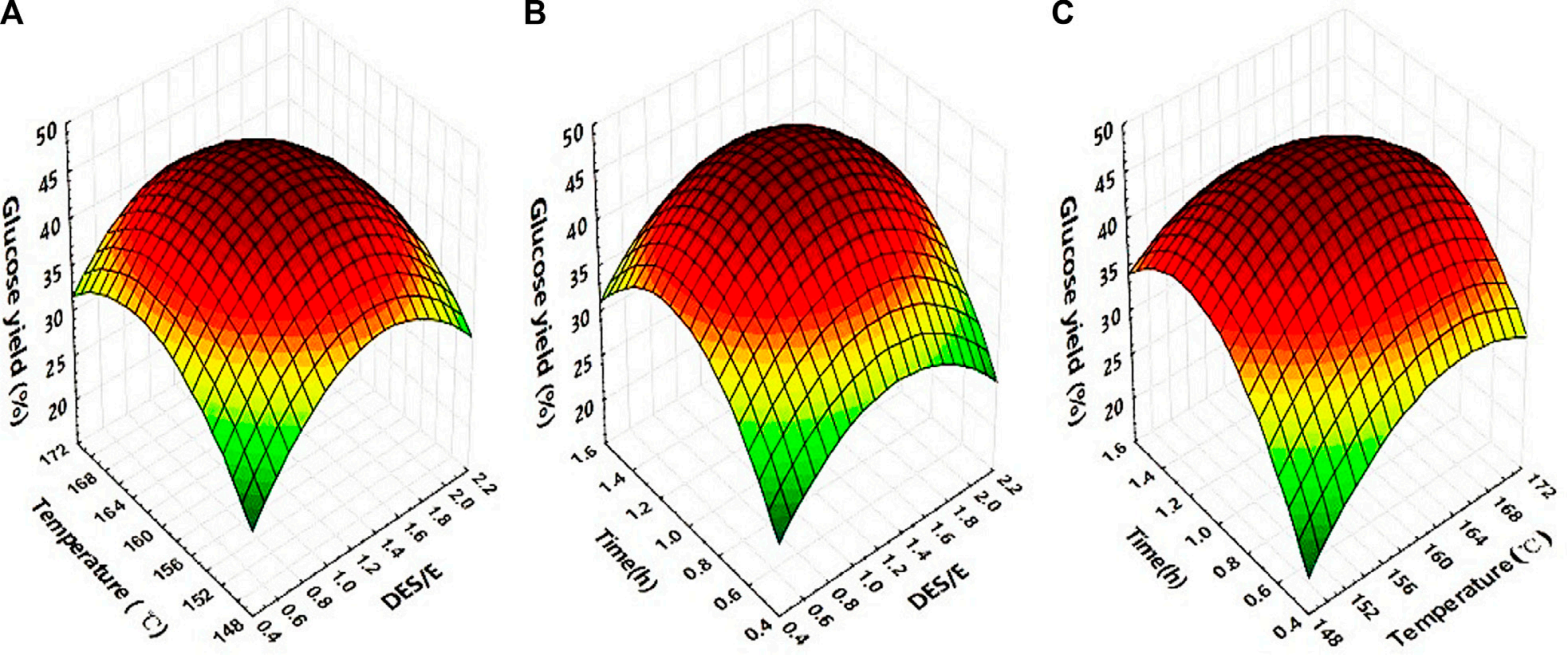
Response surface curve representing the interactive effect of DES to E ratio, temperature and time on digestibility of *Broussonetia papyrifera*: effect of temperature and DES to E ratio **(A)**. Effect of DES to E ratio and time **(B)**. Effect of temperature and time **(C)**.

The highest glucose yield (46.25%) was in trials No 15, which was 130.67% higher than that of control. Figure 2 was the response surface curves based on the interaction between mol ratio of DES to E, temperature and time, taking glucose yield as the response value.

Table 2 was the variance analysis of the glucose yield of the three studied factors. From the results, it was obvious to see that the regression of this model was extremely significant (*p* < 0.0001). The determination coefficient *R* ^2^ was 0.9830, indicating that the linear relationship between the dependent variable and the independent variable was significant. Comparing the value of F, the order of factors affecting the glucose yield was: time > DES to E ratio > temperature. The results also showed that the DES to E ratio, temperature and time, as well as the interaction of the three factors, had a very significant effect on the glucose yield (*p* < 0.01).

**TABLE 2:** Analysis of variance of glucose yield.

	Sum of squares	Df	Mean square	F value	*p*-value prob > F
Model	935.86	10	93.59	52.18	<0.0001
A-Ratio of DES to E	22.74	1	22.74	12.68	0.0061
B-Temperature	19.83	1	19.83	11.06	0.0089
C-Time	99.30	1	99.30	55.36	<0.0001
AB	16.90	1	16.90	9.42	0.0134
AC	0.074	1	0.074	0.041	0.8439
BC	52.33	1	52.33	29.17	0.0004
A^2^	43.55	1	43.55	24.28	0.0008
B^2^	34.21	1	34.21	19.08	0.0018
C^2^	107.33	1	107.33	59.84	<0.0001
*ABC*	61.27	1	61.27	34.16	0.0002
Residual	16.14	9	1.79	—	—
Lack of Fit	16.14	4	4.04	—	—
Cor Total	952.01	19	—	—	—

### 3.2 Component analysis of *Broussonetia papyrifera* after pretreatment

The glucose yield is closely related to the composition of biomass. The influence of different pretreatment conditions on chemical composition of *Broussonetia papyrifera* was shown in Table 3. The content of cellulose, xylan and lignin in untreated raw materials was 36.31%, 23.89%, and 19.99%, respectively. After pretreatment, the cellulose content was increased and that of xylan and lignin was decreased at various extent. This may be because during the pretreatment of DES-E, the chemical linkages between hemicellulose and lignin was cleaved, resulting in removal of hemicellulose and lignin, retaining most of the cellulose, which is consistent with the previous researches (Chen et al., 2019; Li et al., 2022).

**TABLE 3:** Component analysis of *Broussonetia papyrifera* after different pretreatment conditions.

	Component (%)			Component removal (%)		
	Cellulose	Xylan	Lignin	Cellulose	Xylan	Lignin
Untreated	36.31	23.89	19.99	—	—	—
1	47.01 ± 1.20	13.65 ± 0.25	15.96 ± 0.12	9.16 ± 0.08	59.24 ± 0.09	43.05 ± 0.24
2	52.55 ± 1.31	11.53 ± 0.42	12.77 ± 0.13	9.94 ± 1.17	69.42 ± 0.34	59.52 ± 0.17
3	61.69 ± 0.01	8.25 ± 0.31	5.73 ± 2.74	14.08 ± 0.43	82.06 ± 0.10	85.11 ± 1.46
4	65.46 ± 3.79	4.68 ± 0.35	4.76 ± 1.07	18.25 ± 1.24	90.79 ± 0.13	88.8 ± 0.54
5	63.80 ± 0.70	6.19 ± 0.83	6.74 ± 0.39	16.72 ± 0.63	87.31 ± 0.31	83.49 ± 0.29
6	69.91 ± 0.25	3.30 ± 0.72	5.80 ± 1.40	20.87 ± 0.47	94.08 ± 0.29	87.56 ± 0.63
7	73.42 ± 0.55	3.02 ± 1.48	4.28 ± 0.41	17.94 ± 0.97	94.68 ± 0.65	91.00 ± 0.22
8	74.10 ± 0.01	0.44 ± 0.35	2.88 ± 0.11	22.37 ± 0.12	99.27 ± 0.14	94.26 ± 0.04
9	63.33 ± 2.94	6.50 ± 1.58	9.07 ± 0.72	9.98 ± 1.21	85.7 ± 0.80	76.15 ± 0.45
10	67.81 ± 0.77	5.31 ± 0.61	4.91 ± 0.55	16.75 ± 0.70	89.76 ± 0.21	88.69 ± 0.19
11	50.52 ± 1.32	14.30 ± 0.11	14.86 ± 0.72	14.61 ± 1.49	62.22 ± 0.59	53.09 ± 1.14
12	68.50 ± 1.23	5.39 ± 2.60	3.43 ± 0.18	17.76 ± 0.93	89.81 ± 1.3	92.25 ± 0.16
13	55.77 ± 1.00	9.60 ± 0.19	11.48 ± 0.48	15.08 ± 0.76	77.13 ± 0.13	67.32 ± 0.24
14	66.48 ± 1.70	4.69 ± 0.54	4.14 ± 0.39	18.35 ± 0.21	90.92 ± 0.31	90.42 ± 0.22
15	63.73 ± 1.98	6.42 ± 2.95	5.73 ± 0.31	15.82 ± 0.62	86.71 ± 1.43	85.83 ± 0.18

Trials No. 9, 15, and 10 were conducted with the same pretreatment temperature and time, the molar ratio of DES to E was 1/2, 1/1 and 2/1, respectively. The removal of cellulose (9.98%, 15.82%, and 16.75%), xylan (85.7%, 86.71%, and 89.76%) and lignin (76.15%, 85.83%, and 88.69%) was increased with the increase of DES to E ratio. DES promotes the cleavage of C-O bond, which favors the dissolution of lignin. However, during pretreatment lignin might re-polymerize with polysaccharide degradation products (such as furfural) to produce pseudo lignin (Li and Zheng, 2017). DES (ChCl/OA/EG) was synthesized to pretreat birch, in which the degree of lignin condensation increased with the increased OA amount (Liu et al., 2021). Therefore, when the DES content increased, the lactic acid content increased accordingly, resulting in enhanced repolymerization of lignin, which made the removal rate of lignin decreased.

*Broussonetia papyrifera* were pretreated at 150°C, 160°C, and 170°C with the same DES to E ratio and time in schemes 11, 15, and 12. The removal of cellulose (14.61%, 14.61%, and 17.76%), xylan (62.22%, 86.71%, and 89.81%) and lignin (53.09%, 85.83%, and 92.25%) were increased with higher pretreatment temperature. The results indicated that high temperature was in favor for breaking the chemical linkage between cellulose, hemicellulose and lignin, thus the removal of hemicellulose and lignin. However, earlier research showed that pseudo lignin could form under high temperature, which leads to rising in lignin content and a decrease in lignin removal rate (Li et al., 2022).

Schemes 13, 15, and 14 were conducted with the same DES to E ratio and temperature for 0.5, 1, and 1.5 h. The removal of cellulose (15.08%, 15.82%, and 18.35%), xylan (77.13%, 86.71%, and 90.92%) and lignin (67.32%, 85.83%, and 90.42%) were all increased with the extension of time. Earlier study also showed that solubility of pretreated beech by multiple groups of DES was increased with the extension of pretreatment time (Mamilla et al., 2019).

In conclusion, when the DES to E ratio, temperature and time increased independently, the removal of cellulose, hemicellulose and lignin increased. The removal of cellulose was much less than that of hemicellulose and lignin. When the DES to E ratio, temperature and time were increased simultaneously, the changes of chemical composition were the most significant. Pretreatment condition of Scheme 8 was conducted at DES to E of 2:1, 170°C for 1.5 h. The cellulose content in the solid residue was 74.10% and 0.44% xylan and 2 .88% lignin. The cellulose removal of this scheme was relatively high (22.37%), which was not in favor of higher glucose yield.

### 3.3 Factors affecting cellulose conversion of Broussonetia papyrifera

**FIGURE 3:**
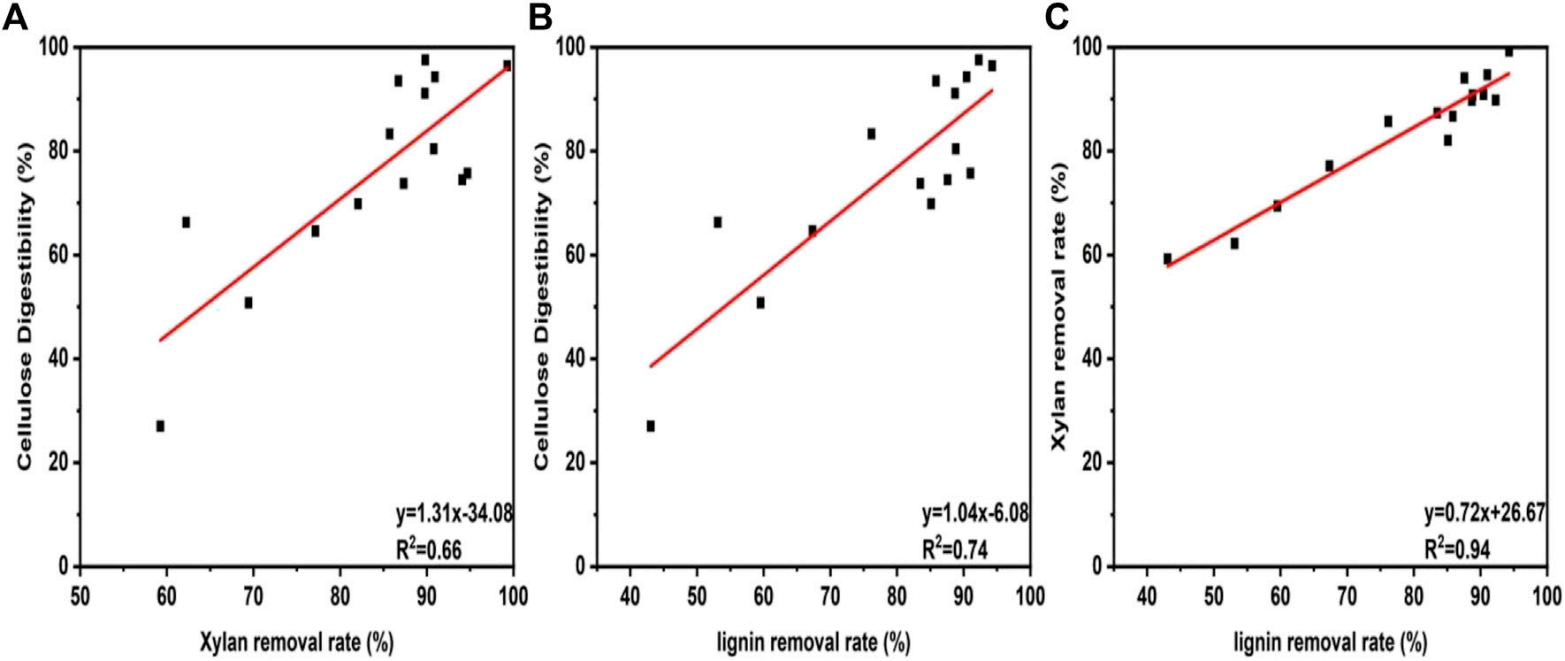
Relationship between xylan removal rate and cellulose digestibility **(A)**. Relationship lignin removal rate and cellulose digestibility **(B)**. Relationship between lignin removal rate and xylan removal rate **(C)**.

From the component analysis, it can be observed that DES-E has a strong ability in biomass fractionation. In order to determine whether the improvement of cellulose digestibility is due to the removal of xylan and lignin during pretreatment, the correlation between their removal and cellulose digestibility were evaluated, as shown in Figure 3. It was observed that xylan removal (*R* ^2^ = 0.66, Figure 3A) and lignin removal (*R* ^2^ = 0.74, Figure 3B) showed great impact on enzyme digestibility of pretreated *Broussonetia papyrifera*. In addition, linear correlation between lignin removal and xylan removal was also found (*R* ^2^ = 0.94, Figure 3C). The results indicated that the removal of xylan and lignin had a positive effect on cellulose conversion to glucose. The removal of lignin exhibited a more obvious effect than that of xylan. Earlier studies showed that removal of xylan can improve the accessibility of enzyme to cellulose surface, thus promoting cellulase hydrolysis (Martínez et al., 2015). In addition, our previous research results showed that there was a non-productive binding between lignin and cellulase. Removal of lignin could make more free cellulase for cellulose degradation (Yao et al., 2022). Bamboo residues were pretreated with DES by different molar ratios of choline chloride/lactic acid by Lin and his coworkers (Lin et al., 2020). The results showed that the xylan and lignin removal were positively correlated with the cellulose degradation, which was consistent with our research results.

### 3.4 Physical property changes after pretreatment

**FIGURE 4:**
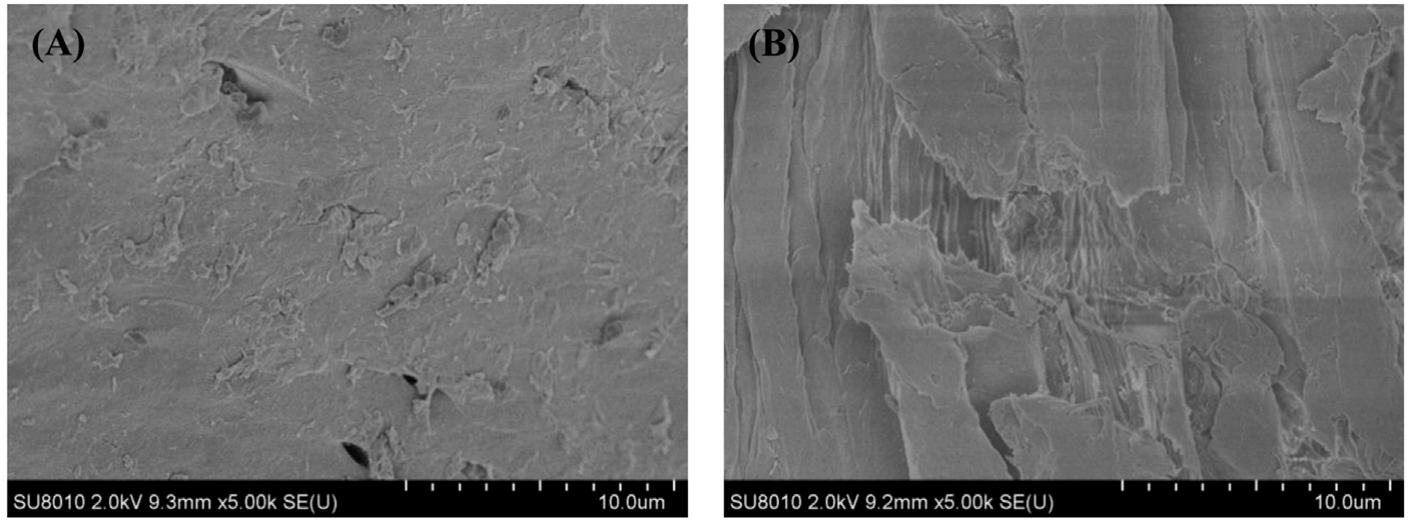
SEM images of *Broussonetia papyrifera* before pretreatment **(A)**. After pretreatment at the best conditions **(B)**.

The SEM images of the samples before and after pretreatment by DES-E at were shown in Figure 4. SEM images of untreated *Broussonetia papyrifera* showed a compact and dense cell wall with complete surface (Figure 4A). However, it can be observed that the structure was loose and rough after DES-E pretreatment. In addition, cracks were observed on the pretreated samples obviously (Figure 4B). The chemical linkages between cellulose, hemicellulose and lignin was destroyed during DES-E pretreatment, removing a large amount of xylan and lignin, leaving residue rich in cellulose (Li et al., 2022). Therefore, due to its rough fiber surface after pretreatment with DES-E, the accessibility of cellulose was improved, thus promoting cellulose digestibility. SEM image analysis after DES pretreatment of beech and corn strover showed that the surface is rougher and looser (Zhao et al., 2018; Mamilla et al., 2019), which was also found in the present study.

**FIGURE 5:**
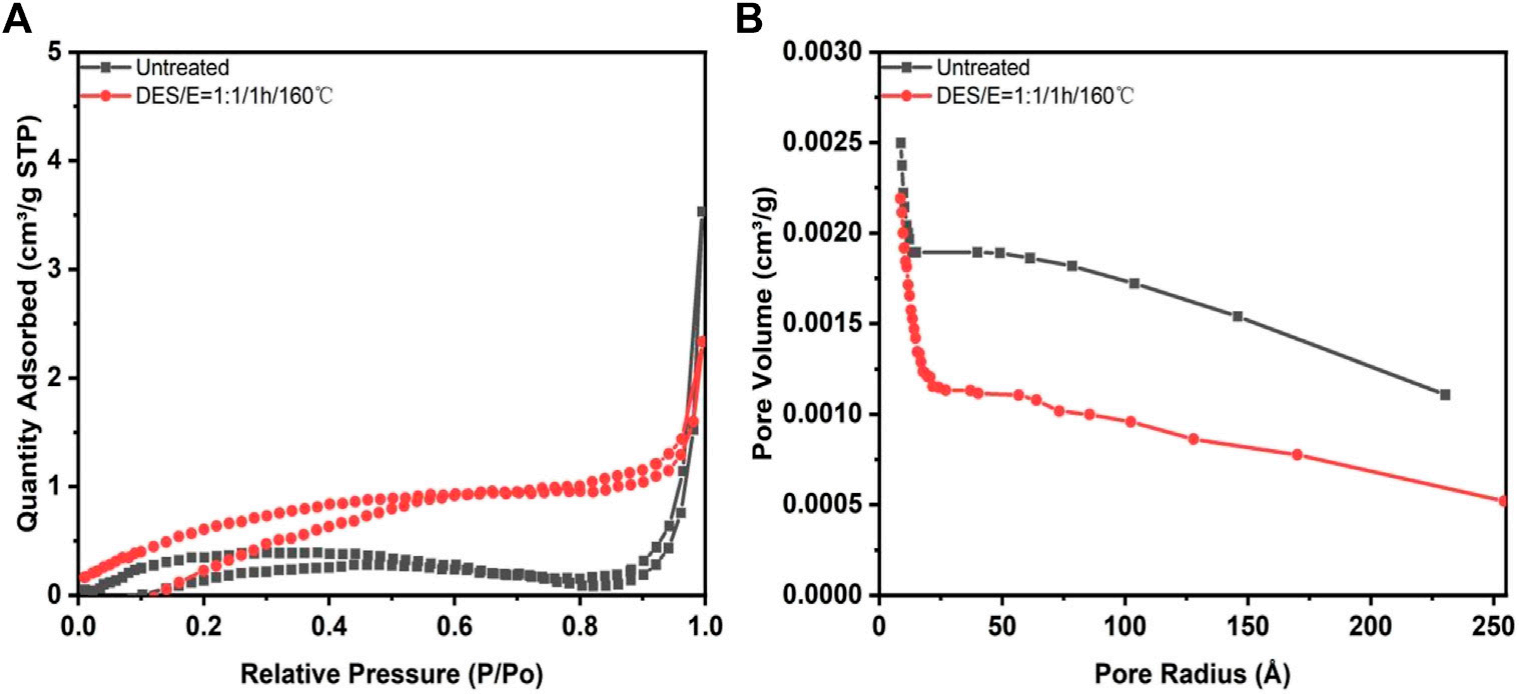
Nitrogen adsorption desorption isotherm curve **(A)**. And BJH pore size distribution diagram **(B)**. Of *Broussonetia papyrifera* before and after pretreatment.

The average pore size and specific surface area are the two most commonly applied parameters to evaluate the pore structure of porous materials (Maloney and Paulapuro, 1999). SEM analysis showed that DES-E pretreatment had a certain impact on the fiber structure. To further explore the impact, average pore size and specific surface area changes after pretreatment was determined, as shown in Figure 5 and Table 4.

**TABLE 4:** Parameters of *Broussonetia papyrifera* before and after pretreatment.

Sample	S_BET_ (m^2^ g^−1^)	Pore volume (cm³ g^−1^)	*Surface area
			Density (m^2^ cm^−3^)
Untreated	1.5987	0.005456	293
After pretreatment	2.6691	0.003538	754

Note: *Surface area density, S_BET_/Pore volume.

It was showed in Figure 5A that both *Broussonetia papyrifera* was non-porous structured polymer before and after pretreatment (Altwala and Mokaya, 2020). When the relative pressure is less than 0.3, the nitrogen adsorption capacity of *Broussonetia papyrifera* after pretreatment was higher than that of the untreated one, indicating that the surface area was increased after pretreatment. Porous materials were classified into three categories according to the pore size: micropores (radius < 2 nm), mesopore (radius <50 nm); macropore (radius >50 nm). It can be seen from Figure 5B that the wood fibers before and after pretreatment showed obvious double pore distribution characteristics. After pretreatment, more microporous structures were formed, which is beneficial to the cellulase hydrolysis. Results in Table 4 indicated that the pore volume after pretreatment was relatively reduced, but the BET surface area and surface area density were increased. BET surface area was increased from 1.5987 to 2.6691 m^2^/g after pretreatment. In an early study, bamboo was pretreated with DES composed by choline chloride, oxalic acid and ethylene glycol. After pretreatment, the surface area was increased significantly (Li et al., 2022). The increase of specific surface area can improve the accessibility of cellulose, so as to achieve high cellulose digestibility and promote the increase of glucose yield.

### 3.5 Physiochemical characteristic analysis of recovered lignin

**FIGURE 6:**
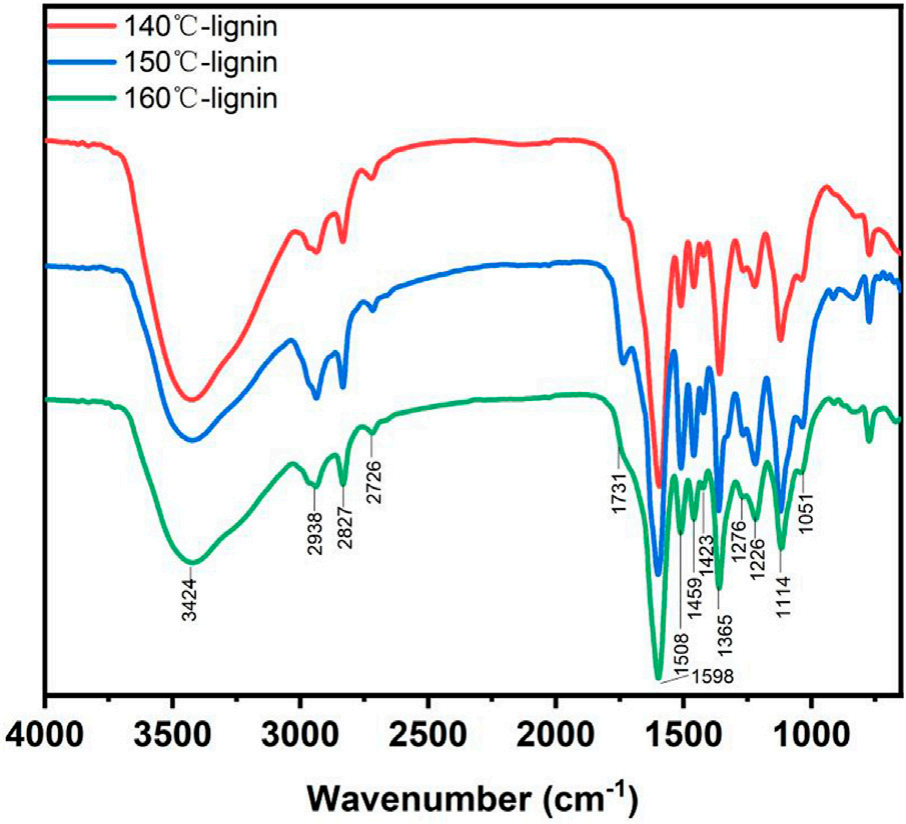
FTIR of recovered lignin after DES-E pretreatment.

FTIR is a widely employed method to analysis different functional groups of lignin. A comparison between recovered lignin at different temperatures showed that the common property and signals in the fingerprint region of the three lignin samples were quite similar. The assignment of important signals in FTIR was referred to previous studies (Yang et al., 2016; 2020). As shown in Figure 6, signals at 3424 cm^−1^ were attributed to the stretching vibration of hydroxyl group (O-H) in lignin. The absorption bands at 2938 cm^−1^ was assigned to the C-H stretching. Signals centered at 1731 cm^−1^ was from the carbonyl stretching not conjugated with the aromatic ring. Strong signals at 1598, 1509, and 1423 cm^−1^ corresponding to aromatic rings were clearly observed in all lignin samples. Furthermore, crosspeak at 1365cm^−1^ was corresponded to syringyl and condensed guaiacyl units. Signals at 1226cm^−1^ was assigned to the C-O absorption of guaiacyl based units. Therefore, the recovered lignin from *Broussonetia papyrifera* pretreated by DES-E was composed by guaiacyl and syringyl.

The ratio of relative signal intensity of different functional groups to that at 1509 cm^−1^ was shown in Table 5 (Yao et al., 2018b). By comparing the relative signal intensity of the three recovered lignin, it can be seen that signal from carbonyl not conjugated with the aromatic ring centered at 1731 cm^−1^ was not found in recovered lignin at 160°C, indicating that C=O in the lignin structure was destroyed at higher pretreatment temperature. The intensity of signals at 1051 cm^−1^ was the strongest in recovered lignin at 150°C. The signal peaks at 1126 and 1114 cm^−1^ were corresponded to guaiacyl-type and syringyl-type lignin, respectively. Comparing the three lignin samples, the S/G of 140°C- lignin was significantly higher than that of the other two lignin samples.

**TABLE 5:** Signal assignment and relative intensities of recovered lignin at different temperatures.

Assignment	Wavenumber (cm^−1^)	140°C-lignin	150°C-lignin	160°C-lignin
Hydroxyl group	3424	1.84	0.90	1.13
C-H stretching	2938	0.63	0.78	0.81
C=O in unconjugated ketone	1731	0.42	0.71	-
Aromatic ring	1598	3.24	1.50	1.82
Aromatic ring	1509	1.00	1.00	1.00
C-H deformation	1459	0.86	0.96	0.93
Aromatic ring	1423	0.67	0.85	0.83
Syringyl and condensed guaiacyl	1365	1.66	1.21	1.31
C-O stretching	1226	0.83	1.00	0.93
Aromatic C-H deformation in syringyl	1114	1.26	1.18	1.06
C-O-C stretching	1051	0.78	0.89	0.77

Note: the relative intensity was calculated as the ratio of the intensity of the signal to the intensity of the band at 1,509 cm.

The molecular weight analysis could elucidate the variations in depolymerization/repolymerization reactions of lignin during pretreatment. GPC method was employed to determine and compare the weight average molecular weight (M_w_), number average molecular weight (M_n_) and polydispersity index (PDI) of each lignin sample. As shown in Table 6, M_n_ of lignin sample recovered in pretreatment liquid decreased with the increase of temperature, which was consistent with the previous research results (Yao et al., 2020a). M_w_ was increased with the increase of temperature, indicating of enhanced lignin repolymerization occurring with increased temperature during acid-catalyzed pretreatment process (Yao et al., 2018). Earlier studies showed that the extensive repolymerization reactions took over with increasing temperature, forming larger and heterogeneous lignin macromolecules (Wang et al., 2012), which could explain the increased M_w_ and PDI with the increased pretreatment temperature in the present study.

**TABLE 6:** Molecular weight and phenolic OH distribution of recovered lignin samples.

	Mn	Mw	Mw/Mn	Phenolic OH (mmol/g)
140°C-lignin	1352	2731	2.02	0.21 ± 0.01
150°C-lignin	948	3311	3.49	0.22 ± 0.01
160°C-lignin	749	3835	5.12	0.40 ± 0.06

The results in Table 6 showed that the phenolic hydroxyl content in the recovered lignin increased with the increase of temperature. This may be due to the broken of α, β-ether linkage during pretreatment, resulting in an increase in phenolic hydroxyl group (Wang et al., 2020). When the pretreatment temperature was at 160°C, the content of phenolic hydroxyl group content was 0.40 mmol/g, as twice as that at 140°C. The result of -OH group content determined by FT-IR and UV was contradictory. The possible reason was that UV method was employed to determine the phenolic hydroxyl group, while in the FTIR, the signal around 3400 cm^-1^ was assigned to total hydroxyl group. The least signal intensity of lignin recovered at pretreatment temperature of 160°C might be caused by less content of aliphatic -OH group (Yao et al., 2020). Earlier studies showed that with the increase of temperature, the content of phenolic hydroxyl group of lignin obtained by hydrothermal pretreatment increased by 53.79% (Chen et al., 2016), which was consistent with our research results. More phenolic OH groups is a desired feature for potentially application of lignin as antioxidant, as reported by other researches previously (Sadeghifar et al., 2017; Yao et al., 2018a).

### 3.6 Antioxidant activity determination of recovered lignin

**FIGURE 7:**
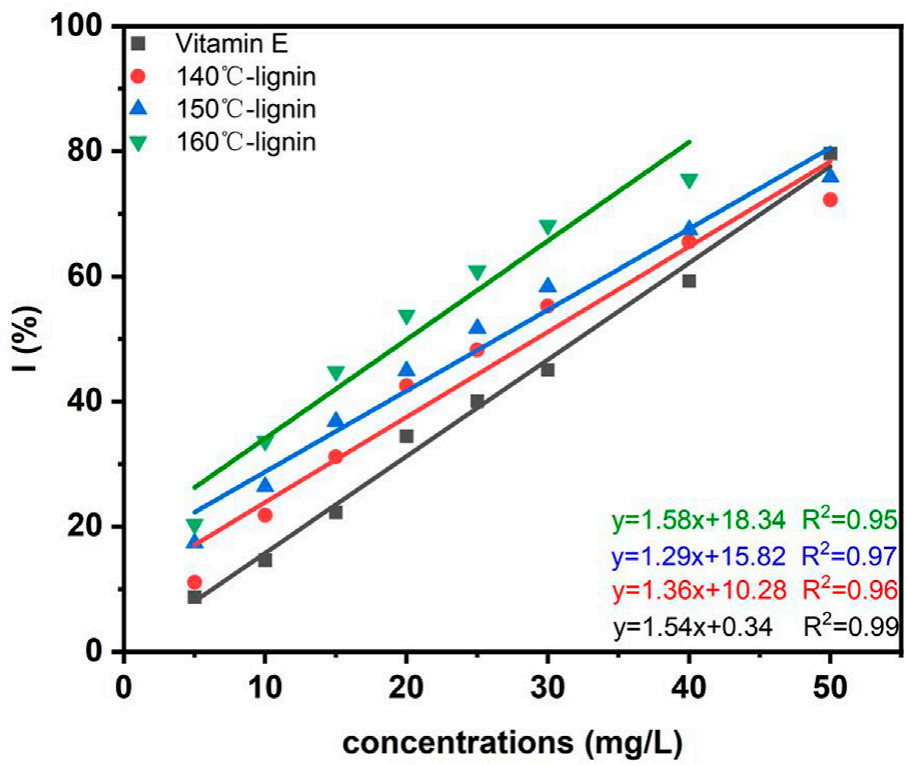
Isotherm of DPPH radical scavenging rate of recovered lignin.

Lignin is a natural aromatic polymer composed of three phenylpropane structural units (*p*-hydroxyphenyl (H), guaiacyl (G) and syringal (S)) connected by various ether bonds and C-C bonds (Sadeghifar and Argyropoulos, 2015). As a compound rich in active functional groups, lignin can capture and neutralize free radicals and shows good antioxidant activity (Lu et al., 2022). In the present study, the *in vitro* antioxidant activity of lignin recovered from the DES-E pretreatment liquid was determined by DPPH method as described earlier (Sadeghifar et al., 2017). The DPPH radical scavenging rates of lignin extracted at different temperatures and water-soluble vitamin E were shown in Figure 7. In the range of 0–50 mg/L, with the increase of lignin concentration, the free radical scavenging capacity of recovered lignin and water-soluble vitamin E was enhanced, and the concentration of lignin is linearly correlated with the free radical scavenging rate (*R* ^2^ ≥ 0.95). The IC_50_ and antioxidant activity index (AAI) were shown in Table 7. IC_50_ represents the concentration of antioxidant required to inhibit 50% free radicals under study. The lower IC_50_ indicates the stronger antioxidant activity. The results showed that compared with vitamin E, the antioxidant activity of recovered lignin pretreated by DES-E was higher. More importantly, the IC50 of these lignin samples was lower than most of published studies (An et al., 2017; Yao et al., 2018b; Dong et al., 2020), indicating advantage of DES-E pretreatment in producing lignin with higher antioxidant activity.

**TABLE 7:** Parameters of antioxidant activity of recovered lignin by DPPH method.

	Linear equation	*R* ^2^	IC_50_ (mg/L)	AAI
Vitamin E	y = 1.55x + 0.34	0.99	32.14	0.77
140°C-lignin	y = 1.36x + 10.28	0.96	29.17	0.84
150°C-lignin	y = 1.29x + 15.82	0.97	26.40	0.93
160°C-lignin	y = 1.58x + 18.34	0.95	20.04	1.23

With the increase of pretreatment temperature, the broken of ether linkage and C-C bond was increased (Li et al., 2022), and the content of phenolic hydroxyl group was increased, which favored the capture of free radicals by lignin. The antioxidant properties of industrial lignin from different sources was studied earlier, and it was found that the phenolic hydroxyl group content in lignin structure was positively correlated with AAI (y = 0.797x + 0.99, *R* ^2^ = 0.978), and the phenolic hydroxyl in lignin played a crucial role in the antioxidant properties (Sadeghifar et al., 2017). The antioxidant activity of lignin sample is mainly from their ability to donate H and quench the free radical intermediate, which is primarily from free phenolic hydroxyl groups. That could explain the importance of the phenolic hydroxyl groups in the antioxidant capability of lignin sample. In another research, it was indicated that the carbonyl group in the side chain inhibited the free radical scavenging activity (Dizhbite et al., 2004). Combined with the FTIR analysis results, there was no obvious signal from C=O in recovered lignin at 160°C, which led to the improvement of its antioxidant capacity. Furthermore, the S/G of the three lignin samples were 1.52, 1.18 and 1.27, respectively, based on the FTIR analysis. It was indicated before that AL with a higher S/G ratio had stronger antioxidant activities (Gong et al., 2016), which was not observed in the present study. However, it was found that lignin with higher Mw and PDI showed higher antioxidant ability, which was contradictory with previous study (Yao et al., 2018b). It might due to limited data in this study. The impact of molecular weight and distribution of lignin on its antioxidant capacity needs further investigation.

## 4 Conclusion

*Broussonetia papyrifera* was pretreated with DES-E to improve the glucose yield and obtain the recovered lignin for further application. When the ratio of DES to ethanol was 1:1, the pretreatment temperature was 160°C, and time was 1h, the highest glucose yield was 46.25%. The physical and chemical properties of the pretreated solid residue and dissolved lignin were analyzed. It was found that compared with the untreated one, the content of lignin and hemicellulose decreased significantly. The specific surface area was also increased after pretreatment. With the increase of temperature, the content of phenolic hydroxyl of recovered lignin was increased, which was beneficial to the antioxidant capacity.

## Data Availability

The original contributions presented in the study are included in the article/Supplementary Material, further inquiries can be directed to the corresponding authors.
